# Supplementary material for: An integrated model of threshold-based scaling and fractional admission controlling to improve resource utilization efficiency in 5G core networks
Source: PLoS One. 2025 Aug 18;20(8):e0330072. doi: 10.1371/journal.pone.0330072 (PMC12360521; doi:10.1371/journal.pone.0330072)
Supplement: Attachment — Submitted filename: Response to Reviewers.pdf [file pone.0330072.s001.pdf]

Dear Mr/Mrs,

First, we would like to thank you for your critical comments. We respectively explain the editor and the reviews as follows. Changes and additions are highlighted in yellow.

1. **The editor**

No.	Comments	Explanations
1	Please ensure that your manuscript meets PLOS ONE's style requirements, including those for file naming.	We have reviewed and revised according to the journal's format and named the files as requested.<br>- Response to Reviewers.<br>- Revised Manuscript with Track Changes.<br>- Manuscript.<br>- Cover Letter.
2	Thank you for stating the following in the Acknowledgments Section of your manuscript:<br>"This work was supported by the Ministry of Education and Training (Vietnam) for the 362 development of Science and Technology under grant number B2023-DHH-17."<br>We note that you have provided funding information that is not currently declared in your Funding Statement. However, funding information should not appear in the Acknowledgments section or other areas of your manuscript. We will only publish funding information present in the Funding Statement section of the online submission form.<br>Please remove any funding-related text from the manuscript and let us know how you would like to update your Funding Statement. Currently, your Funding Statement reads as follows:<br>"The author(s) received no specific funding for this work."<br>Please include your amended statements within your cover letter; we will change the online submission form on your behalf.	We would like to update the content of our Funding Statement in the online submission system to read: "This work was supported by the Ministry of Education and Training (Vietnam) for the development of Science and Technology under grant number B2023-DHH-17." Please update the online submission form on our behalf.
3	We note that your Data Availability Statement is currently as follows:<br>"All relevant data are within the manuscript and its Supporting Information files."	We have added the data as requested by the journal.

No.	Comments	Explanations
	Please confirm at this time whether or not your submission contains all raw data required to replicate the results of your study. Authors must share the "minimal data set" for their submission. PLOS defines the minimal data set to consist of the data required to replicate all study findings reported in the article, as well as related metadata and methods (https://journals.plos.org/plosone/s/data-availability#loc-minimal-data-set-definition).<br><br>For example, authors should submit the following data:<br><br>- The values behind the means, standard deviations and other measures reported;<br><br>- The values used to build graphs;<br><br>- The points extracted from images for analysis.<br><br>Authors do not need to submit their entire data set if only a portion of the data was used in the reported study.	

2. **The reviewer 1**

No.	Comments	Explanations
1	Explicitly address how this model can adapt to varying traffic loads and user behaviors in heterogeneous environments	According to the definition of the traffic load $\rho = \lambda / (k\mu)$, where k is the number of servers. Based on the parameter values in the paper, $k \leq C \times L = 640$. In addition, for the system to operate stably, we need the condition $\rho \leq 1$ or $\lambda / \mu \leq 640$. Therefore, we choose to simulate and analyze with the value $\lambda / \mu \leq 500$ as appropriate. In the case of $\lambda / \mu > 500$, we can infer from the case of $\lambda / \mu \leq 500$ as shown in Figures 5, Figures 6, Figures 13, Figures 14, Figures 15, and Figures 16.<br><br>As for user behavior, here we consider the case of multiple users or multiple UE user devices, where each UE user device can request to establish one or more PDU sessions in the system.<br><br>**We have updated at the lines from 311 to 316:** "*According to the definition of the traffic load* $\rho = \frac{\lambda}{k\mu}$, *where* k *is the number of servers. Based on the*

No.	Comments	Explanations
		parameter values in the paper, we deduce $k \leq C \times L = 640$. *For the system to operate stably, we need the condition* $\rho \leq 1$ *or* $\frac{\lambda}{\mu} \leq 640$. *Therefore, the value* $\frac{\lambda}{\mu} \leq 500$ *is appropriately chosen to simulate and analyze. In addition, assuming that there are multiple UE and each UE can request the establishment of multiple PDU sessions.*”
2	There is no explicit mention of data repositories or links for external data availability, which may be necessary for full compliance with PLOS data policies. Then you have to include raw simulation data or repository links for transparency and replication	We have added the data as requested, including the analysis data and simulation data.
3	Review for minor grammatical errors and consistency in technical terminology. For example, standardize terms like “UPF instance” and “Pod” where necessary	We have reviewed and agreed to add the terminology, with “UPFs” being changed to “UPF instances”. As for the terms “UPF instances” and “Pods”, we have decided to retain them as they are. The reason is that “UPF instances” is a general term used to describe the number of UPFs in both simulation and analysis, while “Pods” refers to the virtualization function of “UPF instances” for simulation in Kubernetes.<br><br>**We have replaced the term “UPFs” with “UPF instances” in Abstract and at the lines: 19, 46, 65-66, 267-268, 268, 273, 283, 284-285, and 366.**<br><br>**We have updated at the line 290:** “*it can be referred to as an UPF Pod in Kubernetes.*”<br><br>**We have corrected some spelling and grammar mistakes, including:**<br><br>- “*Specifically, the need to optimize unused UPF instances, to reduce system costs*” was corrected to “*Specifically, the need to optimize unused UPF instances to reduce system costs*” **in Abstract**.<br>- “*PDU sessions provides*” was corrected to “*PDU sessions provide*” **at the lines 8 and 9**.<br>- “*the operation of the UPF instance are performed by*” was corrected to “*the operation of UPF instances are performed by*” **at the lines 17 and 18**.<br>- “*A TS-FAC algorithm is developed and implemented in Kubernetes-based Open5GS*”

No.	Comments	Explanations
		was corrected to "*A TS-FAC algorithm has been developed and implemented in Kubernetes-based Open5GS*" **at the lines 26 and 27**.<br>- "*operation costs*" was corrected to "*operational costs*" **at the line 48**.<br>- "*dynamic multi-level model, where the thresholds change automatically, and the*" was corrected to "*dynamic multi-level model, where the thresholds change automatically and the*" **at the line 60**.<br>- "H_1 *and* H_2 *are independent of the deploying threshold* T_1 *and the terminating threshold* T_2" was corrected to "H_1 *and* H_2 *are independent of the deployment threshold* T_1 *and the termination threshold* T_2" **at the lines 170 and 171**.<br>- "*we conducted simulations and compared results from 100 to 300 simulations, obtaining average results as shown in Figs 5 and 6*" was corrected to "*we conducted 100 to 300 simulations and compared the averaged results, as shown in Figs 5 and 6*" **at the lines from 296 to 298**.<br>- "*in Section*" was corrected to "*in the Section*" **at the line 305**.<br>- "*distribution resources*" was corrected to "*distributed resources*" **at the line 368**.
4	Enhancing figure captions with detailed descriptions would improve their standalone interpretability. So add Figures captions	We have reviewed Figures and added additional information as requested.<br>**We have added additional information to Figure 1, Figure 3, Figure 4, Figure 7, Figure 8, Figure 9, and Figure 10.**

3. **The reviewer 2**

No.	Comments	Explanations
1	The literature review includes relevant references but could be further enriched with recent studies on optimization and machine learning in resource management for 5G networks. I recommend incorporating the following references to align the manuscript with current advancements in the field:<br><br>https://doi.org/10.1016/j.eswa.2023.122147	**We have added the reference https://doi.org/10.1016/j.eswa.2023.122147 at the line 195:** "*By applying the addition and multiplication rules, as well as the algorithmic complexity calculation methods as in [25]…*"

No.	Comments	Explanations
	https://doi.org/10.21608/jaiep.2024.386693<br>https://doi.org/10.54216/JAIM.080103<br>These citations will enhance the manuscript's contextual relevance and support the discussion on optimization and system performance.	
2	FAC Threshold Selection: Elaborate on the criteria for selecting H_1 and H_2 thresholds and their impact on system performance.	We performed a comparison and evaluation with the range $\|S\|/10$ in Figures 7 and 8 (where $\|S\|$ is the total number of system states). The results show that when $H_2 \leq 7\|S\|/10$, the number of idle UPF instances remains almost unchanged at around 0.45 (the lowest value). However, when $H_2 > 7\|S\|/10$, the number of idle UPFs gradually changes according to the value of H_2, with the exception of the case where $H_1 = H_2 = \|S\|$, which corresponds to the Q-TSUPF model in [2], yielding the highest number of idle UPF instances. In terms of performance, the results remain stable at a level above 0.9921, or 99,21%. Additionally, we have chosen the default values of $H_1 = \|S\|/3$ and $H_2 = 2\|S\|/3$, which signify an even distribution and random selection.<br><br>**We have updated at the lines from 322 to 328:** "*The results show that when $H_2 \leq 7\|S\|/10$, the number of idle UPF instances remains almost unchanged at around 0.45 (the lowest value). However, when $H_2 > 7\|S\|/10$, the number of idle UPF instances gradually changes according to the value of H_2, with the exception of the case where $H_1 = H_2 = \|S\|$, which corresponds to the Q-TSUPF model in [2], yielding the highest number of idle UPFs. In terms of performance, the results remain stable at a level above 0.9921, or 99,21%, which demonstrates the suitability of our proposed theoretical model.*"
3	Computational Efficiency: Discuss the computational complexity of the TSUPF-FAC algorithm, particularly in large-scale deployments with high traffic loads.	With the parameters as specified in the paper, the complexity of the algorithm can be determined in parts as:<br>• Initializing $I(t) = 0$: $O(1)$.<br>• Initializing $J(t) = M$: $O(1)$.<br>• Assigning $N_{J(t)} = J(t) \times C$: $O(1)$.<br>• Assigning the default values $\beta_{I(t),J(t)} = 1$: $O(1)$.<br>• Checking conditions to assign whether $\beta_{I(t),J(t)} = \alpha_1$ or $\beta_{I(t),J(t)} = \alpha_2$: $O(1)$.

No.	Comments	Explanations
		• Setting arrival rates according to $\beta_{I(t),J(t)}$: $O(1)$.<br>• Receiving incoming requests according to $\beta_{I(t),J(t)}$: $O(1)$.<br>• Checking the condition for scaling-out an UPF instance: $O(1)$.<br>• Terminating an in-service request: $O(1)$.<br>• Checking the condition for scaling-in an UPF instance: $O(1)$.<br>• Executing the algorithm loops: $O(C \times L)$.<br>By applying the rules of addition and multiplication, we deduce that the algorithm's complexity is $O(C \times L)$. Furthermore, if the system's scale increases, meaning C or L increases, the complexity will grow linearly since the algorithm's complexity is $O(C \times L)$ (a first-degree polynomial of C or L). The traffic load increase causes the system to reach a full state (i.e., unable to accept new requests) faster and does not affect the algorithm's complexity.<br><br>**We have updated at the lines from 194 to 197:** "*By applying the addition and multiplication rules, as well as the algorithmic complexity calculation methods as in [25], we deduce that the algorithm's complexity is $O(C \times L)$. From the algorithmic complexity, we can see that it depends linearly on C and L, and is independent of traffic load.*"
4	Real-World Applicability: Provide insights into how the proposed model could be integrated into existing 5G networks and its adaptability to future 6G architectures.	Our model is simulated using the core network with Open5GS and integrated into Kubernetes, where the management and allocation of UPF instances are controlled by a Controller. These Controllers can monitor resources in Kubernetes, such as Pods, ReplicaSets, Deployments, StatefulSets, DaemonSets, etc., and take actions to adjust them when thresholds T_1 and T_2 are reached. The Controller in Kubernetes operates similarly to OAM (Operations, Administration, and Management) in the 5G network system. The 6G network is a successor and development of the 5G network with significantly higher speeds and AI integration to optimize the network in real time. In the 6G network, the Mobility Management Function (MMF) is analogous to the AMF in the 5G network, the Communication Management Function (CMF) is similar to the SMF in the 5G network, and the Communication Node (CN) is similar to the UPF in

No.	Comments	Explanations
		the 5G network. Therefore, integrating the TS-FAC algorithm into the 6G network is feasible.<br>**We have updated at the lines from 371 to 372:** "*A crucial aspect is that our model can be applied in 6G networks.*"
5	Comparative Analysis: Expand the discussion on how the proposed model outperforms other state-of-the-art scaling and admission control techniques in terms of efficiency and scalability.	In our paper, we have conducted an evaluation and comparison with the results from [2]. The results show that the model we proposed, Q-TSUPF-FAC, outperforms the Q-TSUPF model from [2]. In the future, some machine learning methods, such as reinforcement learning and deep reinforcement learning, will be considered for integration into the algorithms to further enhance the model's performance as well as to potentially expand the model's applicability to various environments.

We hope our explanations have answered your comments.

Best regards,
