# Supplementary material for: An integrated model of threshold-based scaling and fractional admission controlling to improve resource utilization efficiency in 5G core networks
Source: PLoS One. 2025 Aug 18;20(8):e0330072. doi: 10.1371/journal.pone.0330072 (PMC12360521; doi:10.1371/journal.pone.0330072)
Supplement: Attachment — Submitted filename: Response to Reviewers_R2_Final.docx [file pone.0330072.s002.docx]

Dear Mr/Mrs,

First, we would like to thank you for your critical comments. We respectively explain the editor and the reviews as follows. Changes and additions are highlighted in yellow.

1. **The editor**

| **No.** | **Comments** | **Explanations** |
| --- | --- | --- |
| 1 | Please include the following items when submitting your revised manuscript. | We have reviewed and revised according to the journal’s format and named the files as requested.  - Response to Reviewers.  - Revised Manuscript with Track Changes.  - Manuscript.  - Cover Letter. |

2. **Reviewer #1**

| **No.** | **Comments** | **Explanations** |
| --- | --- | --- |
| 1 | While the manuscript mentions some practical considerations for real-world applications, a more detailed discussion of potential limitations (e.g., impact of network dynamics on performance) would strengthen the paper and provide insights into areas for future improvement. | In our model, we assume a static network environment with arrival rates and service rates for PDU session requests, which is a common simplification in theoretical studies, and we have provided parameter values taken from [2]. However, we acknowledge that network dynamics in real-world systems can significantly impact the performance of the model, and we aim to provide a more detailed discussion of this issue.  **Impact of Network Dynamics on Performance**   1. **Fluctuations in traffic patterns:** In real-world networks, traffic patterns are highly variable and influenced by factors such as user mobility, application demand, and network congestion. The arrival rates of PDU session requests may not always follow a Poisson distribution, as assumed in our model. Instead, traffic may exhibit burstiness, long-range dependence, or periods of heavy congestion that can lead to queueing delays and packet losses. These fluctuations can significantly degrade the performance of the network, affecting key metrics such as throughput, latency, and packet drop rates. 2. **Heterogeneity in network resources:** Networks are often heterogeneous, with varying capacities across different access technologies (e.g., LTE, 5G, Wi-Fi) and network infrastructure (e.g., core vs. edge network). The performance of our model may be slightly influenced if these heterogeneous resources are not considered, particularly regarding the allocation and management of different network slices or frequency bands. In practical scenarios, network performance may vary depending on load balancing, resource contention, and interference between different network segments, which could affect the QoS during the establishment and maintenance of PDU sessions. 3. **Network congestion and load balancing:** Dynamic congestion, where certain parts of the network experience higher traffic volumes than others, can influence the ability of the network to establish or maintain PDU sessions. Load balancing algorithms must continuously adjust to the network’s changing conditions to avoid bottlenecks. In real-time, if the algorithm fails to adapt effectively to congestion spikes or traffic shifts, session establishment delays or session failures could occur, leading to reduced network efficiency and QoS degradation. Our algorithm TSFAC has accounted for idle resources to ensure that a sudden surge in incoming traffic does not cause congestion. 4. **Mobility and user behavior:** In mobile networks, user mobility is another significant factor. Users constantly move between cells or access networks, and the network must adapt to this mobility to maintain stable connections. A model that assumes static conditions might not capture the effects of handovers, changing signal strengths, or cell overloads. These factors can impact the time taken to establish PDU sessions, the continuity of sessions, and even session reliability, especially in high-mobility scenarios like vehicles or public events. 5. **Environmental interference and external factors:** In many practical settings, external interference (such as radio frequency interference, weather conditions, or physical obstructions) can also affect the performance of the network. These dynamic factors can cause fluctuations in network availability and quality, leading to disruptions in service that could affect session establishment and resource allocation.   **Addressing Network Dynamics in Future Work**  To better capture these real-world challenges, we plan to extend our model by incorporating non-Poisson traffic models, such as the General distribution (G) or Erlang distribution (E_k_), to simulate bursty traffic and congestion scenarios more accurately. Furthermore, we will explore adaptive load balancing mechanisms and mobility models that account for the dynamic behavior of users and network resources in heterogeneous environments. This will allow us to evaluate the model’s resilience and performance under varying levels of network load and mobility.  Additionally, as previously mentioned, we will incorporate machine learning (ML) techniques to predict and adjust to network dynamics in real-time. ML can help model the time-varying nature of traffic and network conditions, enabling dynamic optimization of resources, congestion management, and traffic prediction. This approach can also help us automatically tune the parameters of the model based on real-time data, improving its adaptability to network fluctuations in practical scenarios.  **We have updated at the lines from 371 to 374:** “To fulfill the requirements in heterogeneous environments, we will consider applying random processes other than Poisson processes. Additionally, artificial intelligence and machine learning approaches will also be considered to improve computational results in heterogeneous networks.”. |

We hope our explanations have answered your comments.

Best regards,
