# Supplementary material for: An integrated model of threshold-based scaling and fractional admission controlling to improve resource utilization efficiency in 5G core networks
Source: PLoS One. 2025 Aug 18;20(8):e0330072. doi: 10.1371/journal.pone.0330072 (PMC12360521; doi:10.1371/journal.pone.0330072)
Supplement: Attachment — Submitted filename: Response to Reviewers.docx [file pone.0330072.s003.docx]

Dear Mr/Mrs,

First, we would like to thank you for your critical comments. We respectively explain the editor and the reviews as follows. Changes and additions are highlighted in yellow.

1. **The editor**

| **No.** | **Comments** | **Explanations** |
| --- | --- | --- |
| 1 | Please include the following items when submitting your revised manuscript. | We have reviewed and revised according to the journal’s format and named the files as requested.  - Response to Reviewers.  - Revised Manuscript with Track Changes.  - Manuscript.  - Cover Letter. |

We thank the reviewers for this observation

2. **The reviewers**

| **No.** | **Reviewer #3** | **Explanations** |
| --- | --- | --- |
| 1 | On lines 33 – 140 there is information about the use of a model based on a Poission distribution of session arrivals. This has already been risen in the previous comments by other reviewers, but I think the response added to the paper is not sufficient. The references to the literature or an analysis should be provided showing if the session arrivals in 5G have a Poisson distribution or how far from the Poisson distribution they are. | We greatly appreciate your interesting comment and would like to respond as follows.  In our model, it is assumed that the PDU session arrival rate follows a Poisson distribution, while the service rate follows an exponential distribution - an approach consistent with the assumptions in [2][12], among others. Furthermore, simulations have been carried out to verify the practical feasibility of the proposed model.  Nevertheless, in future studies, we intend to extend our evaluation by employing alternative stochastic processes—such as the G (General), Eₖ (Erlang), and D (Deterministic) processes—to model non-Poisson traffic arrivals. This effort is aimed at demonstrating the versatility of our model and ensuring its adaptability to varying traffic conditions, particularly in heterogeneous network environments.  **We have updated at the lines from 140 to 144:** “In fact, the assumption of Poisson-distributed session arrivals in 5G networks has been introduced in [2],[12],[25][26]. Poisson distribution is often used to model events such as incoming calls, network connection requests, or data sessions (e.g., PDU sessions in 5G networks). Non-Poisson-distributed arrivals also exist in 5G networks, but this paper only focuses on Poisson-distributed arrivals.”.  **We have updated at the lines from 310 to 314:** “In our model, it is assumed that the PDU session arrival rate follows a Poisson distribution, while the service rate follows an exponential distribution - an approach consistent with the assumptions in [2],[12], among others. Furthermore, simulations have been carried out to verify the practical feasibility of the proposed model.”. |
| 2 | The figures 5 and 6 don’t add much to the paper. They show that the simulation results are consistent for different seeds, which is good, but this could be shown more clearly by adding the confidence intervals on the plots or listing them in the table in the paper. Instead of comparing the simulations to simulations, the model should be validated by comparison of results from Open5GS testbed with the results obtained using Markov chain model. Has such validation for the Q-TSUPF-FAC been made? | We acknowledge these valuable suggestion and have revised our work accordingly, as detailed below.  Illustrative data have been included in Figs 5 and 6 to confirm the convergence of our proposed algorithm. It is also worth noting that the Open5GS-based experiment represents only one means of modeling the core network. For theoretical analysis, we utilized a Markov chain approach, and the corresponding analytical results are provided in Figs 15 and 16.  **We have updated at the lines from 304 to 306:** “Furthermore, to validate the accuracy of our theoretical model in alignment with practical simulations, the results are presented in Figs 15 and 16.”. |
| 3 | It would be more clear to use the name of the metric in the figures caption, namely the utilization of radio resources instead just U, or the average number of idle instances instead of Vid. | We appreciate these comment and have incorporated the feedback with the following modifications.  Since the y-axis range in some figures was relatively small, we initially labeled them using symbolic notations. However, following Reviewer 3’s suggestion, we have updated the figures accordingly. |
| 4 | The figures 7 and 8 are supposed to compare the Q-TSUPF-FAC and Q-TSUPF algorithms, but it’s very hard to understand from the plots what is the difference. Especially on figure 8, the metric U remains unchanged, regarding the parameters used. Does it mean that the utilization remains the same for both algorithms? Why then the number of idle instances is different? | We acknowledge your engaging perspective and respond as follows.  We offer the following clarification: Although the utilization level remains the same, average number of idle UPF instances is lower under the TS-FAC mechanism because fewer UPF instances are deployed on average, resulting in more efficient resource consumption. For instance, at a utilization level of 0.99, the TS-FAC mechanism results in an average of 0.4 idle UPF instances, whereas the Threshold-based Scaling mechanism results in 0.5. This implies that the TS-FAC mechanism reduces the number of deployed UPF instances by 0.1 on average, highlighting its resource efficiency.  **We have updated at the lines from 338 to 341:** ”As shown in Figure 7, the number of idle UPF instances is lower, but the utilization level does not change. The result suggests that integrating FAC into Q-TSUPF makes the number of UPF instances deployed more efficiently while maintaining the same utilization level.”. |
| 5 | The gain on the better utilization of Q-TSUPF-FAC shown on the figure 12 is very small – it is smaller than 1 percent point. There is too little comment in the paper, why the authors propose to complicate the algorithm for such a small increase of efficiency? | We appreciate your constructive feedback and provide the following explanation.  To emphasize the superiority of our proposed model over that presented in [2], we utilized parameter values comparable to those in [2]. Although the resulting performance improvement is approximately 1%, it is important to note that C=8 in this evaluation, meaning that each UPF can handle up to only 8 concurrent PDU sessions. Consequently, increasing the value of C is expected to yield even more favorable outcomes.  **We have updated at the lines from 358 to 360:** “Although the results in Fig 12 show that the TSFAC algorithm only increases the performance by about 1%, the improvement is noticeable as each UPF can serve more PDU sessions.”. |
| 6 | The conclusions seems to not correctly reflect the content of the paper. It is stated in line 368 that the advantage of the algorithm is the ease of implementation. In my understanding, the proposed method is a slight modification of the previously developed by the same authors Q-TSUPF algorithm. The previous algorithm is less complicated and easier to implement, while the currently proposed Q-TSUPF-FAC offers a slightly better utilization of resources at the cost of using more threshold for the scaling of the load. This should be clearly stated in the conclusions. Additionally, the fact that the model can be applied in 6G networks is both true for the current algorithm and for the previous one. | We are grateful for your insightful opinion and would like to address it as follows.  The algorithm can be readily implemented, as it shares the same complexity as the one presented in [2] ($O\left( C\times L \right)$). Both this model and the previous one can be applied to future 6G mobile networks, although appropriate modifications will naturally be required, since 6G networks are expected to fundamentally retain the user plane functions of the 5G core.  **We have updated at the lines from 386 to 387:** “The computational complexity of the algorithm is $O\left( C\times L \right)$, but it is more efficient regarding resource utilization in fewer idle UPF instances.”. |
| 7 | There is no analysis of the probability of not allocating the sufficient number of UPF instances. I understand that this is caused by the simplification made in the model that the allocation time is equal 0. In such a case the optimal strategy is to allocate the UPF instance as late as possible. A trivial strategy to configure the two thresholds used in the Q-TSUPF algorithm to the maximum possible values should be optimal in such a scenario. Is this a case and why then complicate the algorithm instead of selecting the right thresholds for the already existing method? | We sincerely value your thoughtful input and provide the following reply.  We have ignored the initialization time factor because, during the simulation, the UPF Pod initialization time is extremely small - under 0.1 μs - thus we considered it negligible. It is true that if the initialization time were truly zero, delayed instantiation would be optimal, as it would avoid resource usage until necessary. However, in reality, traffic arrivals are not uniform and may experience sudden spikes. In such cases, delayed instantiation could lead to temporary UPF shortages (before supplementary instances can be launched), resulting in QoS violations. The FAC algorithm - which performs slightly earlier instantiation based on segmentation—helps maintain system stability and prevents performance shocks during sudden traffic surges. Moreover, due to the combination of TS and FAC mechanisms, the risk of under-provisioning the required number of UPF instances is effectively eliminated. |
|  | **Reviewer #4** | We sincerely thank and appreciate your valuable contributions to our manuscript. |
